# Skeletal muscle health in childhood cancer survivors: a systematic review and meta-analysis

**DOI:** 10.1007/s00520-026-10425-3

**Published:** 2026-02-13

**Authors:** Anna Maria Markarian, Dennis R. Taaffe, Daniel A. Galvão, Carolyn J. Peddle-McIntyre, Jodie Cochrane Wilkie, Francesco Bettariga, Nicholas G. Gottardo, Mayank Dhamija, Santosh Valvi, Catriona M. Buchanan, Kerrie Graham, Robert U. Newton

**Affiliations:** 1 Exercise Medicine Research Institute, Edith Cowan University, 270 Joondalup Drive, Joondalup, Western Australia 6027 Australia; 2 School of Medical and Health Sciences, Edith Cowan University, Joondalup, Western Australia Australia; 3 Physical Activity, Sport and Exercise Research Theme, Faculty of Health, Southern Cross University, Gold Coast, Australia; 4 Western Australian Bone Research Collaboration (WABRC), Perth, WA Australia; 5 Department of Paediatric and Adolescent Oncology/Haematology, Perth Children’s Hospital, Nedlands, WA Australia; 6 Brain Tumour Research Program, Telethon Kids Institute, Nedlands, WA Australia; 7 Therapeutic Expertise, Medical Affairs, ICON Biotech, Dublin, Ireland; 8 Division of Paediatrics, School of Medicine, The University of Western Australia, Perth, Western Australia Australia; 9 University of Notre Dame Australia, Fremantle, Western Australia Australia; 10 School of Human Movement and Nutrition Sciences, University of Queensland, St. Lucia, QLD 4072 Australia

**Keywords:** Childhood cancer, Muscle quantity, Muscle quality, Muscle function, Meta-analysis, Systematic review

## Abstract

**Purpose:**

Childhood cancer survivors (CCS) are at risk of long-term skeletal muscle deficits following intensive therapies during critical periods of growth. This review aimed to synthesize approaches for assessing muscle quantity, quality, and function in CCS and to quantify deficits relative to healthy peers.

**Methods:**

A systematic search was conducted in CINAHL, Embase, PubMed, SPORTDiscus, and Web of Science from inception to June 2024, with an update in November 2025. Studies including CCS who had completed cancer treatment and reported measures of muscle quantity, quality, or physical function were eligible. A three-level mixed-effects model meta-analysis was conducted. Associations between muscle quantity and function and potential moderators were tested using meta-regression models. Methodological quality was assessed using the Newcastle–Ottawa Scale.

**Results:**

Forty-four studies comprising 5175 CCS were included. Compared to controls, CCS exhibited significantly lower muscle quantity (SMD −0.45; 95% CI −0.63 to −0.28; *p* < 0.001) and muscle function (SMD −0.41; 95% CI −0.57 to −0.24; *p* < 0.001). No studies evaluated muscle quality. Deficits in muscle function were more pronounced in the lower body than the upper body, and meta-regression analyses indicated greater muscle quantity deficits with increasing time since treatment completion.

**Conclusion:**

Childhood cancer survivors exhibit significant deficits in muscle quantity and function. Future research should focus on developing international consensus guidelines for standardized, clinically meaningful assessments that include lower-body function and muscle quantity. Additionally, investigating muscle quality may lead to a better understanding of the mechanisms underlying these deficits and help inform targeted interventions aimed at preserving long-term health in CCS.

**Supplementary Information:**

The online version contains supplementary material available at 10.1007/s00520-026-10425-3.

## Introduction

Childhood cancer survivors (CCS) face substantial long-term physiological challenges that can compromise physical function, independence, and quality of life [1, 2]. As survival rates continue to improve, understanding and mitigating these late effects has become a research priority [1, 3, 4], particularly given the evidence that many CCS experience health deficits decades earlier than the general population [1, 2, 5]. Phenotypic markers of accelerated aging, such as frailty and sarcopenia, have emerged as key indicators for understanding and potentially mitigating quality-of-life impairments in CCS [2, 3, 5]. Traditionally developed for geriatric populations, these constructs now describe physical vulnerability in young adult CCS, who display early parallels with age-related decline [1, 2]. Evidence from the St. Jude Lifetime cohort highlights this trajectory, demonstrating that phenotypic indicators of premature aging in CCS are apparent by age 33 years [1].

Treatment for childhood cancer often coincides with critical periods of musculoskeletal development, when gains in muscle mass and strength typically peak [6]. Exposure to intensive therapies, including neurotoxic chemotherapeutic agents [7] and glucocorticoids [8], during this window can impair muscle development and regenerative capacity [6]. As a result, CCS may experience persistent deficits in muscle quantity, quality (e.g., increased inter- and intramuscular adipose tissue [9]), and function (e.g., reduced strength), which collectively increase the risk of frailty, loss of independence, and adverse health outcomes [6]. In an earlier systematic review and meta-analysis, we identified marked skeletal muscle deficits in children undergoing active cancer treatment [10]. However, among CCS who have completed therapy, evidence remains inconsistent. While Morales and colleagues [11] previously reported no significant deficits in muscle strength or lean body mass, the synthesis relied solely on DXA-derived lean mass and handgrip strength, potentially resulting in an incomplete characterization of skeletal muscle health in CCS.

The assessment and diagnosis of skeletal muscle deficits remain highly heterogeneous, prompting initiatives to develop standardized terminology, diagnostic criteria, and outcome measures [12–14]. Several recent publications have called for harmonization [13, 14], and an international Delphi process is currently underway to define the physically vulnerable CCS and establish consensus-based criteria [3]. In older adults, deficits in muscle quantity and strength (typically handgrip) are commonly assessed [9, 12]. More recently, muscle-specific strength, defined as strength relative to muscle size (e.g., leg extension strength relative to quadriceps volume), has been proposed as an additional indicator of skeletal muscle health [12]. However, the appropriateness and feasibility of applying these indicators to younger populations remain debated [13, 14].

Given the fragmented nature of the evidence, the limited scope of previous syntheses, and the ongoing evolution of consensus definitions, there is a clear need for a comprehensive and contemporary synthesis of the diverse approaches used to assess skeletal muscle health in CCS. To address this gap, we conducted a systematic review and meta-analysis to integrate existing assessment strategies, identify the most significant deficits, and examine moderating factors that may influence skeletal muscle outcomes across the survivorship continuum.

## Methods

A meta-analysis was conducted to investigate skeletal muscle quantity, quality, and function in childhood cancer survivors. Meta-regression models were also used to examine the moderators of these outcomes. All procedures undertaken in this study were reported in accordance with the Cochrane Back Review Group (CBRG) [15], the Preferred Reporting Items for Systematic Reviews and Meta-Analyses (PRISMA) statement (Supplementary Material—Table 1) [16], and the Meta-analysis of Observational Studies in Epidemiology (MOOSE) reporting guidelines [17].

### Search strategy and study selection procedures

This review was registered in the International Prospective Register of Systematic Reviews (PROSPERO) under CRD42024551447. At the time of registration, two review objectives were proposed: (1) to examine the impact of cancer treatment on skeletal muscle quantity, quality, and function in children and adolescents *undergoing active cancer therapy* and (2) to compare these outcomes between childhood cancer survivors *who had completed cancer treatment* and peers with no prior history of cancer. A systematic search was conducted in CINAHL, Embase, PubMed, SPORTDiscus, and Web of Science from database inception to June 08, 2024, with an update in November 05, 2025. The search strategy was undertaken with the assistance of a librarian using controlled vocabulary and free-text terms (Supplementary Material—Appendix 1). The titles and abstracts were independently evaluated by two authors (Anna Maria Markarian and Francesco Bettariga) following the eligibility criteria. Abstracts were selected for full-text evaluation when they did not provide sufficient information. Full-text articles meeting the criteria were retrieved and read independently by both reviewers (Anna Maria Markarian and Francesco Bettariga) and assessed for inclusion in the study. Disagreements regarding the final list of included studies were resolved by consensus.

Given the large number of studies identified and the heterogeneity of included populations, specifically studies involving children undergoing active cancer treatment and those involving childhood cancer survivors following treatment completion, the original study protocol was divided into two separate, focused syntheses to facilitate meaningful interpretation. The first review, which examined skeletal muscle outcomes longitudinally in children *during active treatment*, has been published previously [10]. The present review addresses the second objective by evaluating skeletal muscle quantity, quality, and function in childhood cancer survivors *following treatment completion*, compared to healthy peers.

### Eligibility criteria

The primary outcomes of this review were skeletal muscle quantity, quality, and function. We defined *muscle quantity* as a generic term encompassing lean mass, lean body mass, fat-free mass, and skeletal muscle tissue, given the interchangeable use of these terminologies in the literature. Studies were eligible if they included muscle quantity measures derived from DXA, bioelectrical impedance, D3 creatine dilution, deuterium dilution, or skinfold (in kilograms), as well as regional muscle assessments (e.g., muscle thickness, cross-sectional area, muscle volume) using ultrasound, computed tomography, or magnetic resonance imaging (MRI). *Muscle quality* included measures of muscle composition (e.g., inter-/intramuscular adipose tissue or muscle density) derived from ultrasound, CT, or MRI. *Muscle function* included measures of muscle strength (e.g., isometric or isokinetic tests), muscle power (e.g., jump tests), or muscle endurance (e.g., number of squats performed within a defined period).

To evaluate whether skeletal muscle quantity, quality, and function in CCS differ from children who have not had cancer, we included studies that (a) enrolled survivors of any childhood cancer who were *off treatment*, with no restrictions on the survivors’ age at assessment (the final sample included CCS aged 7–38 years); (b) reported at least one of the endpoints analyzed in this review; and (c) had a control group without a previous history of cancer. There were no restrictions on study design. For intervention studies, only baseline measures prior to any intervention were included. For longitudinal studies, only the latest available time point was extracted.

The exclusion criteria were as follows: (a) studies not reporting outcomes included in this review; (b) studies written in languages other than English; and (c) case reports/series, editorials, abstracts, commentaries, and reviews.

### Data extraction

Data extraction was performed by two authors (Anna Maria Markarian and Francesco Bettariga) using a structured form in the Covidence systematic review platform [18]. The review protocol was embedded in Covidence to ensure that study eligibility was consistently compared against the predefined criteria. Inconsistencies in data extraction were resolved during weekly meetings. Extracted study variables included sample size, study design, cancer diagnosis, age at assessment, height, total body mass and fat, and all outcomes of interest. Where possible, data were extracted as raw means and standard deviations (SD), or in a format that allowed transformation into mean and SD. Three studies reporting muscle quantity outcomes [19–21] and five reporting muscle function outcomes [20, 22–25] provided adjusted means rather than raw values. These adjusted estimates were included in the meta-analysis; however, a sensitivity analysis excluding these studies was performed to show no effect on the overall results. For studies reporting medians, ranges, or interquartile ranges instead of means and SDs, estimates were converted using the method described by Wan et al. [26]. When the standard error (SE) was reported instead of SD, the latter was obtained using the formula from Altman and Bland [27]. For studies that assumed a normal distribution and provided means and confidence intervals (CI), we transformed the CI into SE and then calculated the SD according to the Cochrane recommendations [28]. Finally, when numerical data were only available in graphical format, values were extracted using WebPlotDigitizer (San Francisco, CA) [29].

### Quality assessment

The methodological quality of the included studies was independently assessed by two authors (Anna Maria Markarian and Francesco Bettariga) using the Newcastle–Ottawa Scale (NOS) [30]. Scores range from 0 to 9, with higher scores indicating higher quality (0–3 points low quality, 4–6 points moderate quality, 7–9 points high quality).

### Statistical analysis

A three-level mixed-effects meta-analysis with study included as a random effect was performed to examine skeletal muscle quantity, quality, and function in CCS. Cluster robust point estimates and 95% CIs are reported and weighted by inverse sampling variance to account for the within- and between-study variance (tau^2^). Additionally, a restricted maximal likelihood estimation was used in all models. Statistical significance was assumed when *p* < 0.05. Statistical heterogeneity was assessed using the Cochran *Q* test, with an *I*^2^ greater than 50% indicating high heterogeneity. Publication bias was explored using contour-enhanced funnel plots and Egger’s test [31]. Heterogeneity, publication bias, sensitivity, and moderator analyses were performed to substantiate the results.

For studies reporting muscle quantity, subgroup analyses were conducted by (a) cancer type and (b) assessment modality. For studies reporting muscle function, subgroup analyses were conducted by (a) cancer type, (b) assessment modality, and (c) body region. Multilevel models with robust estimates were generated for each subgroup, and fixed effects with the moderator’s model were used for comparison. Meta-regression models were used to test the association between potential moderators and outcomes of interest. Hypothesized moderators of muscle quantity and function included age at assessment, time off therapy, sex, and study-level mean differences in weight and height relative to controls. Statistical significance was assumed when *p* < 0.05. Analyses were conducted using the meta, metafor, and clubSandwich packages in R (R Core Team, version 4.3.3., 2024).

## Results

**Fig. 1:**
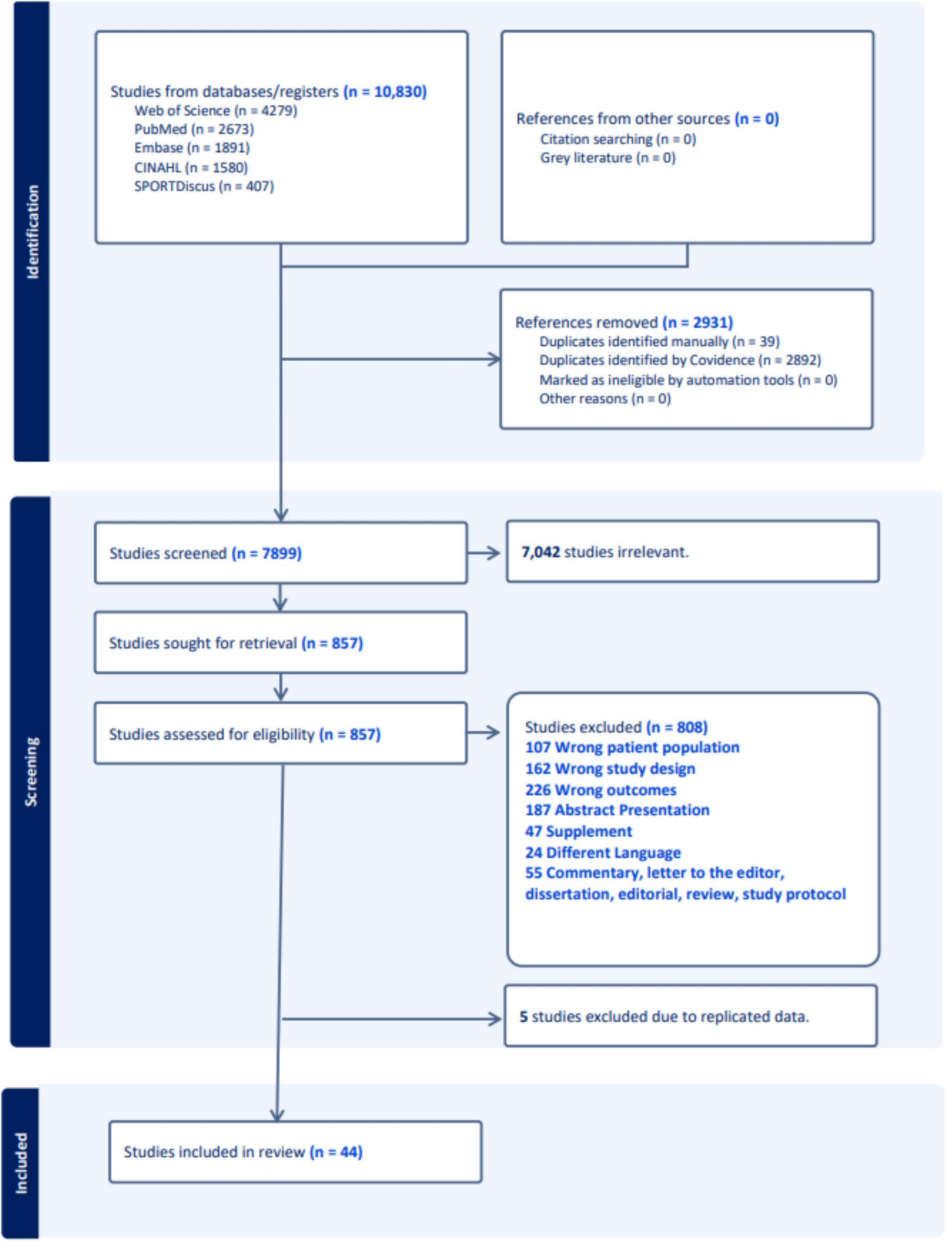
Flow chart of study selection process

A total of 10,830 studies were retrieved from our search. After removing duplicates, 7899 records remained for title and abstract screening. Of these, 7042 were excluded because they were irrelevant to our research question. An additional 808 records were excluded for specific reasons, as detailed in Fig. 1. Finally, five studies (Supplementary Material—Appendix 2) were excluded due to duplicate measurements within the same cohort. As a result, 44 research papers were included in this meta-analysis.

### Study characteristics

Forty-four eligible studies comprising 5175 CCS (mean age 21 years, range 7–38; mean time off treatment 10 years, range 1–20) were identified. Of these, 26 studies assessed muscle quantity [19–21, 32–54], and 21 studies assessed muscle function [20, 22–25, 45, 48, 55–68], with 3 reporting both outcomes [20, 45, 48]. No studies evaluated muscle quality in CCS. A general description of the characteristics of the 44 included studies is provided in Supplementary Table 2, while the risk of bias, assessed using the NOS, is presented in Supplementary Table 3.

### Skeletal muscle outcomes in childhood cancer survivors

#### Muscle quantity

**Fig. 2:**
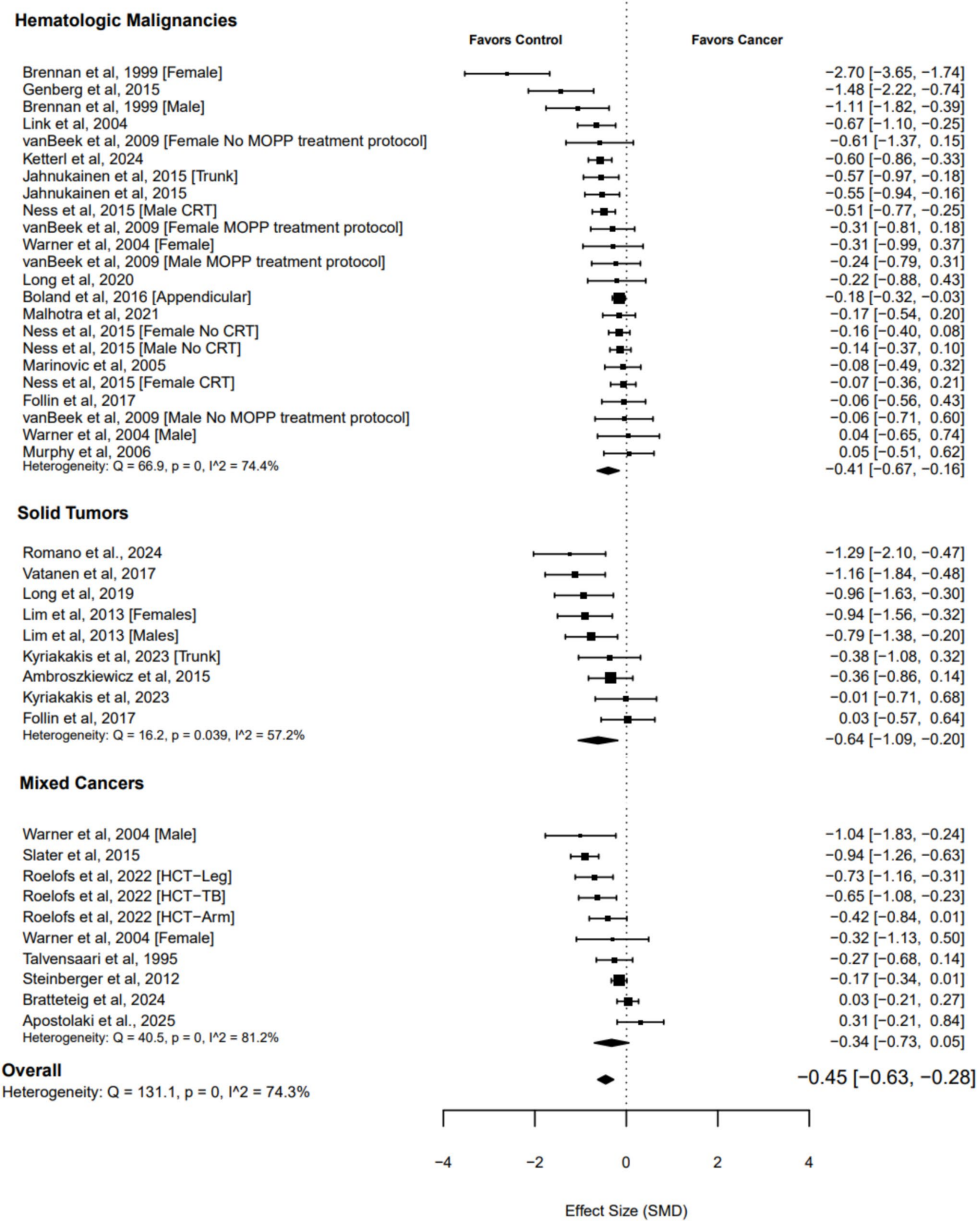
Forest plot of muscle quantity in childhood cancer survivors

The overall pooled standardized mean difference (SMD) was −0.45 (95% CI −0.63 to −0.28; *p* < 0.001; Fig. 2), based on 42 effect sizes across 26 studies [19–21, 32–54]. Heterogeneity was high (*I*^2^ 74.30%), and the contour-enhanced funnel suggested publication bias (*τ* = −2.90; *p* = 0.006; Supplementary Fig. 1).

A complementary analysis of absolute mean differences (20 studies) indicated that DXA-derived total body lean mass was approximately 5.0 kg lower in CCS (95% CI 3.0 to 7.0; *p* < 0.001). These deficits occurred alongside a significantly higher fat mass (+2.5 kg; 95% CI 0.1 to 5.0; *p* = 0.043) and a lower, though statistically nonsignificant, total body mass (−2.5 kg; 95% CI −5.4 to 0.5; *p* = 0.093), suggesting an unfavorable body composition profile in CCS.

Subgroup analyses by cancer type and assessment method (Table 1; Supplementary Table 4) did not indicate either factor as a significant moderator of muscle quantity effect sizes.

**Table 1:** Overall and subgroup analysis of muscle quantity in childhood cancer survivors

	Multilevel random-effects meta-analysis					Heterogeneity	
	*k*	*n*	SMD	95% CI	*p*	*Q*	*I*^2^
Overall effect	26	42	−0.45	−0.63 to −0.28	<0.001	131.10	74.30%
Subgroup analysis by cancer type							
*Hematological malignancies*	14	23	−0.41	−0.67 to −0.16	0.004	66.90	74.40%
*Solid tumors*	7	9	−0.64	−1.09 to −0.20	0.013	16.20	57.20%
*Mixed cancers*	7	10	−0.34	−0.73 to 0.05	0.079	40.50	81.20%
Subgroup analysis by assessment modality							
*DXA*	21	36	−0.50	−0.69 to −0.31	<0.001	117.4	79.30%
*BIA*	3	4	−0.34	−2.26 to 1.58	0.521	11.0	81.20%
*Skinfold*	1	1	−0.27	−0.36 to −0.19	-	-	-
* 4 C model*	1	1	0.05	−0.11 to 0.22	-	-	-

*95% CI* 95% confidence intervals, *SMD* standardized mean difference, *I*^*2*^ within- and between-study heterogeneity, *k* number of clusters, *n* number of effect sizes, *Q* Cochran’s *Q* test of heterogeneity, *DXA* dual-energy X-ray absorptiometry, *BIA* bioelectrical impedance analysis, * 4 C model* 4 compartment model

#### Muscle quality

A meta-analysis for muscle quality could not be performed, as no studies in CCS utilized methods that directly assessed muscle composition. Three studies reported both muscle quantity and function [20, 45, 48]; however, none explicitly quantified the strength-to-mass ratio, a key indicator of muscle quality.

#### Muscle function

**Fig. 3:**
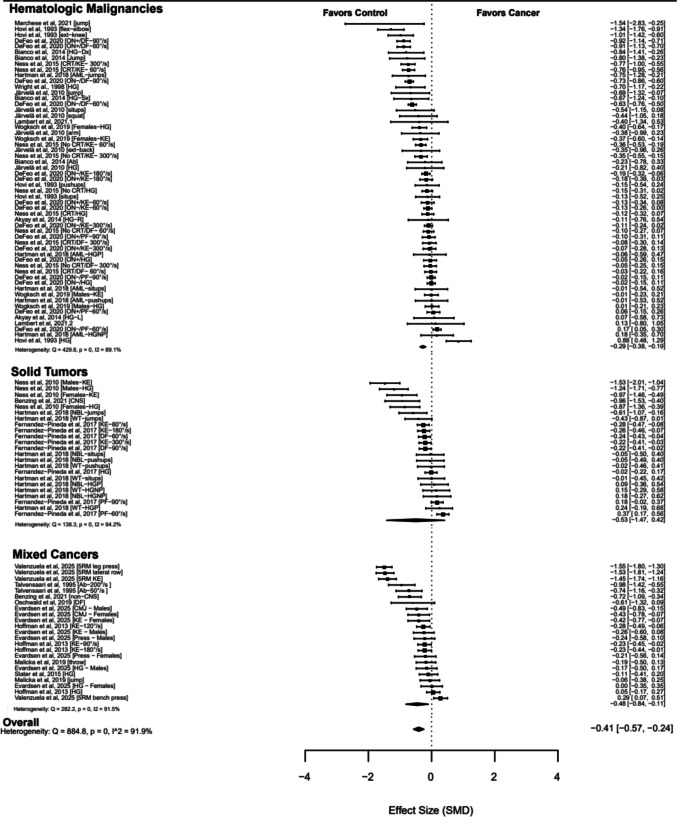
Forest plot of muscle function in childhood cancer survivors

The overall pooled SMD was −0.41 (95% CI −0.57 to −0.24; *p* < 0.001; Fig. 3) based on 102 effect sizes across 21 studies [20, 22, 24, 25, 45, 48, 55–68]. Heterogeneity was high (*I*^2^ 91.90%), and the contour-enhanced funnel plot did not indicate publication bias (*τ* = −1.30; *p* = 0.183; Supplementary Fig. 2).

Subgroup analyses by assessment modality and body region (Table 2; Supplementary Table 4) indicated that deficits were significantly greater for dynamic compared with static assessments (*p* = 0.026) and for the lower body compared with the upper body (*p* = 0.007), reflecting larger reductions in muscle function in these subgroups. Cancer type did not significantly moderate effect sizes.

**Table 2:** Overall and subgroup analyses of muscle function in childhood cancer survivors

		Multilevel random-effects meta-analysis				Heterogeneity	
	*k*	*n*	SMD	95% CI	*p*	*Q*	*I*^2^
Overall effect	21	102	−0.41	−0.57 to −0.24	<0.001	884.80	91.90%
Subgroup analysis by cancer type							
*Hematological malignancies*	11	56	−0.29	−0.38 to −0.19	0.001	429.60	89.10%
*Solid tumors*	4	23	−0.53	−1.47 to 0.42	0.174	138.30	94.20%
*Mixed cancers*	8	23	−0.48	−0.84 to −0.11	0.019	282.20	91.50%
Subgroup analysis by muscle function assessment modality							
*Static strength (isometric assessments)*	14	32	−0.24	−0.44 to −0.04	0.024	144.80	87.10%
*Dynamic strength (isokinetic and functional assessments)*	18	70	−0.44	−0.77 to −0.19	0.005	717.80	92.60%
Subgroup analysis by body region							
*Upper body*	17	46	−0.27	−0.45 to −0.10	0.005	254.02	85.89%
*Lower body*	16	54	−0.53	−0.78 to −0.28	< 0.001	598.65	94.77%

*95% CI* 95% confidence intervals, *SMD* standardized mean difference, *I*^*2*^ within- and between-study heterogeneity, *k* number of clusters, *n* number of effect sizes, *Q* Cochran’s *Q* test of heterogeneity

### Meta-regression analysis of moderators of muscle quantity and function

Muscle quantity (SMD) was significantly negatively associated with time since treatment completion (*β* = −0.05; *p* = 0.016), suggesting that differences in muscle quantity between CCS and controls may widen over time. In contrast, muscle quantity was significantly positively associated with mean differences in height and weight between CCS and controls (height, *β* = 0.06; *p* = 0.002; weight, *β* = 0.06, *p* < 0.001), such that larger deficits in height and weight in CCS relative to controls were associated with larger negative SMDs for muscle quantity. Age at assessment and sex were not significantly associated with muscle quantity (Supplementary Table 5; Supplementary Figs. 3–7).

Muscle function deficits were significantly positively associated with age at assessment (*β* = 0.01; *p* = 0.029). Subgroup analysis by body region indicated that this association was driven by upper-body strength, with older participants exhibiting smaller deficits (*β* = 0.02; *p* = 0.013). Sex, time since treatment completion, and differences in height or weight were not significantly associated with muscle function (Supplementary Table 5; Supplementary Figs. 8–14).

## Discussion

To our knowledge, this is the largest meta-analysis to date synthesizing evidence on skeletal muscle outcomes in childhood cancer survivors. Three key findings emerged. First, childhood cancer survivors exhibit significantly lower muscle quantity and muscle function than healthy peers. Second, deficits in muscle function are more pronounced in the lower body than in the upper body and are more evident in dynamic measures than in static. Third, muscle quantity deficits were significantly associated with clinical and anthropometric factors, with greater deficits linked with longer time since treatment completion and with larger height and weight discrepancies between survivors and controls.

These findings have important clinical implications, given that skeletal muscle deficits in CCS are associated with an increased risk of chronic health conditions [2, 69] and poorer survival [70]. Analysis of differences in muscle quantity between CCS and controls yielded a pooled SMD of −0.45. A complementary analysis using mean differences indicated that total body lean mass was approximately 5 kg lower in the CCS group. For adolescents and young adults at a mean age of 20, a deficit of ~5 kg represents 8–12% of total lean mass. This deficit is clinically meaningful, particularly given that lean tissue accounts for 60–70% of total body mass in healthy young adults [71–73]. We also observed a modest yet significant reduction in muscle function, with an SMD of −0.41.

Morales and colleagues [11] previously reported no significant differences in lean body mass or handgrip strength between CCS and controls in their meta-analysis. However, their synthesis was limited by the small number of included studies, with only eight reporting lean body mass and eight reporting handgrip strength. Moreover, while handgrip strength is a valuable and widely used measure of muscle strength, it primarily reflects upper body isometric strength and does not capture overall muscle function [74]. Lower body strength is essential for daily functioning, underpinning key activities such as ambulation, balance, rising from a chair, and climbing stairs. Large cohort studies have consistently demonstrated substantial functional and performance limitations in CCS [2, 75].

The unique pattern of muscle deficits observed in CCS underscores the need to carefully reconsider how physical vulnerability is defined in this population [3]. Phenotypes of sarcopenia and frailty are widely used markers of vulnerability in older adults. However, their underlying assumptions, particularly the reliance on handgrip strength and age-related physiological decline, may not fully capture the distinct pathophysiology, mechanisms, and patterns of impairment in CCS [3]. Our findings support this distinction, showing potential recovery in upper-body strength, while lower-body deficits appear more pronounced and persistent. Consequently, adult frailty models, such as the Fried criteria [76], which typically assess muscle function via handgrip strength, may not adequately reflect the specific forms of vulnerability experienced by CCS.

Persistent lower-body strength deficits and progressive muscle loss may represent early features of physical vulnerability that contribute to the accelerated aging phenotype frequently described in CCS [2, 5, 75]. We observed a significant negative association between muscle quantity (SMD) and time since treatment completion, suggesting that deficits in muscle mass may increase as survivors age. Impairments in lower-body strength could further exacerbate these deficiencies by accelerating atrophy in large weight-bearing muscles. Because muscle mass and strength are closely linked to metabolic health, such deficits likely contribute to the earlier onset and higher prevalence of chronic health conditions, including cardiovascular and endocrine disorders [34, 69, 77].

Muscle deficits, particularly when accompanied by excess adiposity, increase the risk of cardiometabolic disorders and multimorbidity. In our complementary analyses, muscle deficits co-occurred with significantly higher fat mass, despite no statistically significant differences in total body mass. Consistent with these findings, previous studies have reported a higher fat mass in CCS, but comparable body weight relative to peers [78, 79]. Such imbalances between muscle and fat mass may reflect early metabolic dysregulation, predisposing survivors to cardiometabolic complications later in life. Metabolic impairments may also manifest in altered muscle composition. Notably, no studies assessed muscle quality or muscle-specific strength, measures that could provide mechanistic insight into whether observed functional deficits also arise from compromises in muscle quality, highlighting a critical gap in the literature.

Intensive cancer treatments and radiation therapy can disrupt endocrine function and growth pathways, with downstream effects on musculoskeletal development in CCS [80]. In our analysis, greater deficits in height and weight were associated with larger muscle quantity deficiencies, suggesting that impaired muscle development may reflect broader disruptions in growth and hormonal regulation. While direct treatment-related factors likely contribute to these impairments, indirect effects of cancer and its therapy, such as undernutrition and reduced physical activity during critical growth periods, may also play a role [81, 82]. These findings underscore the importance of early identification of growth faltering as a potential marker of underlying muscle deficits and highlight opportunities for timely interventions during critical developmental windows.

Overall, findings from this study underscore the need for more relevant and standardized assessment of skeletal muscle health in CCS. Standardized measures of functional performance, particularly lower-body strength, could improve clinical relevance. Consequently, frameworks for defining and assessing physical vulnerability in CCS may require careful adaptation to ensure they remain clinically efficient and meaningful. Measures of muscle quantity are also essential, as body weight or BMI alone may not accurately reflect metabolic fitness. Lastly, to better understand underlying mechanisms, future research should investigate muscle quality in CCS. These efforts will guide the development and implementation of appropriate countermeasures. Preventive strategies, such as nutrition and exercise interventions, remain underutilized in CCS [2] despite evidence supporting their feasibility and potential efficacy [83, 84], highlighting the urgent need for high-quality, targeted approaches to address muscle deficits in this population.

Despite the strength of this review, which included 44 studies and 5175 participants to characterize muscle outcomes in CCS, several limitations warrant consideration. First, this meta-analysis was conducted at the study level and did not include individual participant data. Factors such as variation in follow-up duration, evolution of therapeutic protocols, and differences in age or hormonal status may contribute to heterogeneity, with some CCS exhibiting more pronounced deficits than others. Nevertheless, the consistent group-level findings support the presence of clinically relevant impairments in muscle quantity and function, underscoring the importance of routine surveillance for skeletal muscle deficits in CCS. Second, while metabolic disturbances are commonly reported, no studies have assessed muscle quality, precluding a meta-analysis on this outcome. We anticipate that the present findings will serve as a foundation, highlighting this critical gap in the literature. Finally, the mean age of CCS in our dataset was ~21 years, indicating that most participants had not yet reached the age at which the risk of sarcopenia and frailty typically increases. Nevertheless, the presence of muscle deficits at younger ages indicates that age-related muscle loss in this population could be more severe.

## Conclusion

Childhood cancer survivors exhibit significant deficits in skeletal muscle quantity and function compared to healthy peers, with disproportionately greater impairments in lower-body muscle function. Deficits in muscle quantity appear to worsen over time and are associated with disruptions in growth and body composition. Collectively, these findings highlight skeletal muscle as a key component of physical vulnerability in CCS. Standardized, clinically relevant measures, particularly those incorporating lower-body function, muscle quantity, and muscle quality, are urgently needed to improve risk stratification and guide targeted interventions aimed at preserving long-term health in survivors.

## Supplementary Information

Below is the link to the electronic supplementary material.

Supplementary file1 (DOCX 713 KB)

## Data Availability

The data that support the findings of this study are available from the corresponding author upon request.
