# Supplementary material for: Skeletal muscle health in childhood cancer survivors: a systematic review and meta-analysis
Source: Support Care Cancer. 2026 Feb 13;34(3):193. doi: 10.1007/s00520-026-10425-3 (PMC12901291; doi:10.1007/s00520-026-10425-3)
Supplement: Supplementary file 1 — Supplementary file1 (DOCX 713 KB) [file 520_2026_10425_MOESM1_ESM.docx]

Supportive Care in Cancer

**Skeletal Muscle Health in Childhood Cancer Survivors: A Systematic Review and Meta-analysis**

Anna Maria Markarian, MSc^1,2*^; Dennis R. Taaffe, PhD, DSc, MPH^1,2^; Daniel A. Galvão, PhD^1,2^; Carolyn J. Peddle-McIntyre, PhD^1,2^; Jodie Cochrane Wilkie, PhD^1,2,3,4^; Francesco Bettariga, MSc^1,2^; [Nicholas G. Gottardo, MB-ChB](https://www.thelancet.com/journals/lanonc/article/PIIS1470-2045(11)70275-3/fulltext), PhD, FRACP^5,6^; Mayank Dhamija, MD^5,7^; Santosh Valvi, MD^5,6,8^; Catriona M. Buchanan, BMBS, FRACP, FRCPA^5,9^; Kerrie Graham, RN^5^; Robert U. Newton, PhD, DSc^1,2,10^

^1^Exercise Medicine Research Institute, Edith Cowan University, Joondalup, Western Australia, Australia; ^2^School of Medical and Health Sciences, Edith Cowan University, Joondalup, Western Australia, Australia; ^3^Physical Activity, Sport and Exercise Research Theme, Faculty of Health, Southern Cross University, Gold Coast, Australia; ^4^Western Australian Bone Research Collaboration (WABRC), Perth, WA, Australia; ^5^Department of Paediatric and Adolescent Oncology/Haematology, Perth Children’s Hospital, Nedlands, WA, Australia; ^6^Brain Tumour Research Program, Telethon Kids Institute, Nedlands, WA, Australia; ^7^Therapeutic Expertise, Medical Affairs, ICON Biotech, Dublin, Ireland; ^8^School of Medicine, Division of Paediatrics, The University of Western Australia, Perth, Western Australia, Australia; ^9^University of Notre Dame Australia, Fremantle, Western Australia, Australia; ^10^School of Human Movement and Nutrition Sciences, University of Queensland, St. Lucia, QLD 4072, Australia.

*Address for correspondence:

Anna Maria Markarian, PhD candidate, MSc, CSCS

Exercise Medicine Research Institute

Edith Cowan University

270 Joondalup Drive, Joondalup WA 6027, AUSTRALIA

Email: [a.markarian@ecu.edu.au](mailto:a.markarian@ecu.edu.au)

Phone Number: +61 436416366

**LIST OF SUPPLEMENTAL CONTENT**

**Appendix 1** Search Key

**Appendix 2** Exclusions

**Table 1** PRISMA 2020 Checklist

**Table 2** Characteristics of the included studies assessing muscle quantity and function in childhood cancer survivors

**Table 3** Individual risk of bias for studies assessing muscle quantity and function in childhood cancer survivors.

**Table 4** Results of test of moderators for muscle quantity and function in childhood cancer survivors

**Table 5** Meta-regression results for age at assessment, time from diagnosis, cohort year, age at diagnosis, male sex, and height on muscle quantity and function in childhood cancer survivors

**Figure 1** Contour-enhanced funnel plots for overall effects on muscle quantity in childhood cancer survivors

**Figure 2** Contour-enhanced funnel plots for overall effects on muscle function in childhood cancer survivors

**Figure 3-14** Bubble plots of random effects univariable meta-regression for time from diagnosis, age at assessment, sex, study-level mean differences in weight and height on muscle quantity and function. Each circle represents an effect size, and the size of the circle reflects the influence of that study on the meta-regression model

**
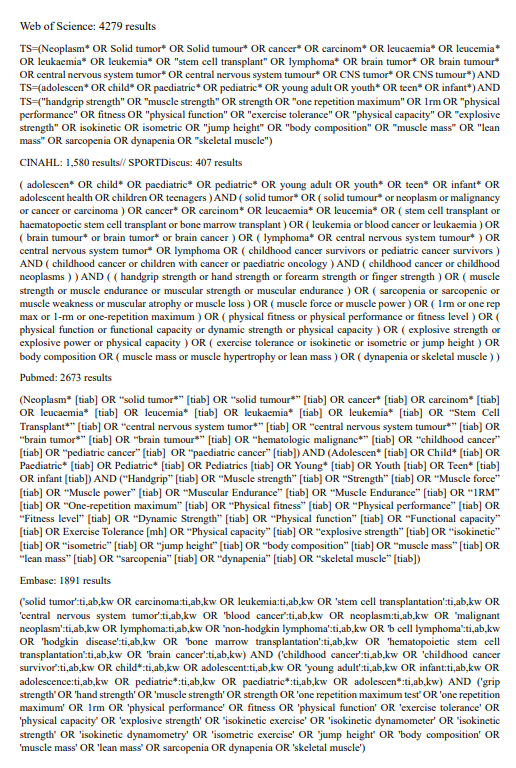
Appendix 1.** Search key**:**

**Appendix 2.** Exclusions

A total of 5 studies were excluded [1-5].

| Author | Publication |
| --- | --- |
| Follin et al. | *Improvement in cardiac systolic function and reduced prevalence of metabolic syndrome after two years of growth hormone (GH) treatment in GH-deficient adult survivors of childhood-onset acute lymphoblastic leukemia.* The Journal of Clinical Endocrinology & Metabolism, 2006. |
| Polgreen et al. | *Modifiable risk factors associated with bone deficits in childhood cancer survivors.* BMC Pediatrics, 2012. |
| Bülow et al. | *Survivors of childhood acute lymphoblastic leukaemia, with radiation‐induced GH deficiency, exhibit hyperleptinaemia and impaired insulin sensitivity, unaffected by 12 months of GH treatment.* Clinical Endocrinology, 2004. |
| Slater et al. | *Physical activity and cardiovascular risk factors in childhood cancer survivors.* Pediatric Blood & Cancer, 2015. |
| Öberg et al. | *Exercise capacity in young adults after hematopoietic cell transplantation in childhood.* American Journal of Transplantation, 2018. |

**Table** 1 PRISMA 2020 checklist

| **Section and Topic** | **Item #** | **Checklist item** | **Location where item is reported** |
| --- | --- | --- | --- |
| **TITLE** | | |  |
| Title | 1 | Identify the report as a systematic review. | 1 |
| **ABSTRACT** | | |  |
| Abstract | 2 | See the PRISMA 2020 for Abstracts checklist. | 2 |
| **INTRODUCTION** | | |  |
| Rationale | 3 | Describe the rationale for the review in the context of existing knowledge. | 3-4 |
| Objectives | 4 | Provide an explicit statement of the objective(s) or question(s) the review addresses. | 4 |
| **METHODS** | | |  |
| Eligibility criteria | 5 | Specify the inclusion and exclusion criteria for the review and how studies were grouped for the syntheses. | 4-5 |
| Information sources | 6 | Specify all databases, registers, websites, organisations, reference lists and other sources searched or consulted to identify studies. Specify the date when each source was last searched or consulted. | 4 |
| Search strategy | 7 | Present the full search strategies for all databases, registers and websites, including any filters and limits used. | Supplementary, Appendix 1, Search Key. |
| Selection process | 8 | Specify the methods used to decide whether a study met the inclusion criteria of the review, including how many reviewers screened each record and each report retrieved, whether they worked independently, and if applicable, details of automation tools used in the process. | 4-5 |
| Data collection process | 9 | Specify the methods used to collect data from reports, including how many reviewers collected data from each report, whether they worked independently, any processes for obtaining or confirming data from study investigators, and if applicable, details of automation tools used in the process. | 4-6 |
| Data items | 10a | List and define all outcomes for which data were sought. Specify whether all results that were compatible with each outcome domain in each study were sought (e.g. for all measures, time points, analyses), and if not, the methods used to decide which results to collect. | 5-6 |
|  | 10b | List and define all other variables for which data were sought (e.g. participant and intervention characteristics, funding sources). Describe any assumptions made about any missing or unclear information. | 6 |
| Study risk of bias assessment | 11 | Specify the methods used to assess risk of bias in the included studies, including details of the tool(s) used, how many reviewers assessed each study and whether they worked independently, and if applicable, details of automation tools used in the process. | 6 |
| Effect measures | 12 | Specify for each outcome the effect measure(s) (e.g. risk ratio, mean difference) used in the synthesis or presentation of results. | 6 |
| Synthesis methods | 13a | Describe the processes used to decide which studies were eligible for each synthesis (e.g. tabulating the study intervention characteristics and comparing against the planned groups for each synthesis (item #5)). | 5 |
|  | 13b | Describe any methods required to prepare the data for presentation or synthesis, such as handling of missing summary statistics, or data conversions. | 6 |
|  | 13c | Describe any methods used to tabulate or visually display results of individual studies and syntheses. | 5-6 |
|  | 13d | Describe any methods used to synthesize results and provide a rationale for the choice(s). If meta-analysis was performed, describe the model(s), method(s) to identify the presence and extent of statistical heterogeneity, and software package(s) used. | 6-7 |
|  | 13e | Describe any methods used to explore possible causes of heterogeneity among study results (e.g. subgroup analysis, meta-regression). | 6-7 |
|  | 13f | Describe any sensitivity analyses conducted to assess robustness of the synthesized results. | 6-7 |
| Reporting bias assessment | 14 | Describe any methods used to assess risk of bias due to missing results in a synthesis (arising from reporting biases). | 6-7 |
| Certainty assessment | 15 | Describe any methods used to assess certainty (or confidence) in the body of evidence for an outcome. | NA |
| **RESULTS** | | |  |
| Study selection | 16a | Describe the results of the search and selection process, from the number of records identified in the search to the number of studies included in the review, ideally using a flow diagram. | 7; Figure 1 |
|  | 16b | Cite studies that might appear to meet the inclusion criteria, but which were excluded, and explain why they were excluded. | 7; Supplementary Material – Appendix 2 |
| Study characteristics | 17 | Cite each included study and present its characteristics. | 7-8; Supplementary Tables 2. |
| Risk of bias in studies | 18 | Present assessments of risk of bias for each included study. | Supplementary Tables 3. |
| Results of individual studies | 19 | For all outcomes, present, for each study: (a) summary statistics for each group (where appropriate) and (b) an effect estimate and its precision (e.g. confidence/credible interval), ideally using structured tables or plots. | 8; Figures 2 and 3. |
| Results of syntheses | 20a | For each synthesis, briefly summarise the characteristics and risk of bias among contributing studies. | Supplementary Tables 2-3. |
|  | 20b | Present results of all statistical syntheses conducted. If meta-analysis was done, present for each the summary estimate and its precision (e.g. confidence/credible interval) and measures of statistical heterogeneity. If comparing groups, describe the direction of the effect. | 8-9; Figures 2 and 3. |
|  | 20c | Present results of all investigations of possible causes of heterogeneity among study results. | 8-9; Tables 1 and 2; Supplementary Figures 1-14. |
|  | 20d | Present results of all sensitivity analyses conducted to assess the robustness of the synthesized results. | Sensitivity analyses were conducted by excluding (i) studies with low NOS scores and (ii) studies reporting adjusted means. |
| Reporting biases | 21 | Present assessments of risk of bias due to missing results (arising from reporting biases) for each synthesis assessed. | 8; Supplementary Figures 1 and 2 |
| Certainty of evidence | 22 | Present assessments of certainty (or confidence) in the body of evidence for each outcome assessed. | 8 |
| **DISCUSSION** | | |  |
| Discussion | 23a | Provide a general interpretation of the results in the context of other evidence. | 9-11 |
|  | 23b | Discuss any limitations of the evidence included in the review. | 11-12 |
|  | 23c | Discuss any limitations of the review processes used. | 11-12 |
|  | 23d | Discuss implications of the results for practice, policy, and future research. | 9-12 |
| **OTHER INFORMATION** | | |  |
| Registration and protocol | 24a | Provide registration information for the review, including register name and registration number, or state that the review was not registered. | 4 |
|  | 24b | Indicate where the review protocol can be accessed, or state that a protocol was not prepared. | 4 |
|  | 24c | Describe and explain any amendments to information provided at registration or in the protocol. | 4 |
| Support | 25 | Describe sources of financial or non-financial support for the review, and the role of the funders or sponsors in the review. | 13 |
| Competing interests | 26 | Declare any competing interests of review authors. | 13 |
| Availability of data, code and other materials | 27 | Report which of the following are publicly available and where they can be found: template data collection forms; data extracted from included studies; data used for all analyses; analytic code; any other materials used in the review. | Available upon request |

*From:*  Page MJ, McKenzie JE, Bossuyt PM, Boutron I, Hoffmann TC, Mulrow CD, et al. The PRISMA 2020 statement: an updated guideline for reporting systematic reviews. BMJ 2021;372:n71.

| Table 2 Characteristics of the studies examining quantity and function in childhood cancer survivors | | | | |
| --- | --- | --- | --- | --- |
| Author, year, country | **Study Design** | **Participant Characteristics (n, sex, cancer type, age in years, height in cm, weight in kg)** | **Control Characteristics (n, sex, age in years, height in cm, weight in kg)** | **Outcome measure included** |
| Hovi et al. 1993,  Finland [59] | **comparative study with a control group** | **43 (0 males) survivors of childhood leukemia; Mean age at study: 19; Height: 159.2; Weight: 54.7** | **69 (0 males); Mean age at study:**  **19.4; Height: 165.7; Weight: 59.5** | **Elbow flexion (n), Knee extension (n), Grip (n), Push-up test (times), Sit-up test (times)** |
| Talvensaari et al. 1995,  Finland [48] | **age and sex-matched cohort study** | **46 (21 males) survivors of childhood cancer (mixed diagnoses); Mean age at study: 19.1; Height: 162.6; Weight: 59.1** | **46 sex-and age-matched; Height: 168.4; Weight: 58.8** | **Fat-free mass (kg) from skinfold,**  **Isokinetic Trunk muscle strength (NG)** |
| Wright et al., 1998,  Canada [62] | **comparative study with a control group** | **36 (25 males) survivors of childhood acute lymphoblastic leukemia; Mean age at study:**  **9.7; Height Z-score: −0.3; Weight Z-score: 0.5** | **36 (25 males); Mean age at study: matched; Height: NG; Weight: NG** | **Handgrip Strength (mmHg)** |
| Rahim et al. 1999,  UK [35] | **comparative study with a control group** | **32 (17 males) survivors of childhood acute lymphoblastic leukemia; Mean age at study: 24.5; Height: NG; Weight: NG** | **35 (18 males); Mean age at study: 22.3; Height: NG; Weight: NG** | **Lean mass (kg) from DXA** |
| Warner et al. 2004,  UK [51] | **comparative study with a control group** | **56 (males NG) survivors of childhood cancer (mixed diagnoses); Mean age at study: 12 (ALL), 11.2 (other cancers); Height: 145.5 (ALL), 143.7 (other cancers); Weight: 49.7 (ALL), 39.8 (other cancers)** | **32 (males NG); Mean age at study: 12.5; Height: 151.8; Weight: 48.6** | **Fat-free mass (kg) from DXA** |
| Link et al. 2004,  Sweden [39] | **comparative study with a control group** | **44 (23 males) survivors of childhood acute lymphoblastic leukemia; Mean age at study: 25.2; Height: 167.3; Weight: 77.4** | **44 (matched with survivors); Mean age at study: matched with patients; Height:**  **176.8; Weight: 75.5** | **Lean mass (kg) from DXA** |
| Marinovic et al. 2005,  France [52] | **Prospective case control study** | **37 (20 males) survivors of childhood acute lymphoblastic leukemia; Mean age at study:**  **10.3; Height: NG; Weight: NG** | **74 (40 males); Mean age at study: matched with survivors; Height: NG; Weight: NG** | **Lean body mass (kg) from DXA** |
| Murphy et al. 2006,  UK [44] | **cross-sectional** | **24 (11 males) survivors of childhood acute lymphoblastic leukemia; Mean age at study: 9.6; Height: 134.7; Weight: 36.9** | **24 (males NG); Mean age at study:**  **9.6; Height: 135.9; Weight: 33.0** | **Fat-free mass (kg) calculated from four-component model** |
| VanBeek et al. 2009,  The Netherlands [49] | **cross-sectional** | **88 (56 males) survivors of Hodgkin's lymphoma; Mean age at study: 28.6; Height: NG; Weight: NG** | **212 (81 males); Mean age at study: NG; Height: NG; Weight: NG** | **Lean body mass (kg) from DXA** |
| Ness et al. 2010,  United States [61] | **comparative study with a control group** | **78 (42 males) survivors of brain cancers; Mean age at study: 22; Height: NG; Weight: NG** | **78 (males NG); Mean age at study:**  **25; Height: NG; Weight: NG** | **Isokinetic knee extension (N), handgrip strength (kg)** |
| Järvelä et al, 2010,  Finland [66] | **comparative study with a control group** | **21 (10 males) survivors of childhood acute lymphoblastic leukemia; Mean age at study: 14.5; Height: 170.7; Weight: 71** | **21 (10 males); Mean age at study: matched; Height:**  **173.2; Weight:**  **70.2** | **Sit-Ups, repeats/60s, Back extensors, repeats/30s, Max vertical jump (cm), Full squatting, repeats/30s, Upper arm, max repeats per side, handgrip strength (N)** |
| Steinberger et al. 2012,  United States [19] | **cross-sectional** | **319 (171 males) survivors of childhood cancer (mixed diagnoses); Mean age at study: 14.5; Height: 158.2; Weight: 57.2** | **208 (112 males); Mean age at study: 13.6; Height:**  **159.9; Weight: 57** | **Lean body mass (kg) from DXA** |
| Hoffman et al. 2013,  United States [24] | **cross-sectional/case-control** | **183 (97 males) survivors of childhood cancer (mixed diagnoses); Mean age at study:**  **13.5; Height: 158.1; Weight: 55.9** | **147 (74 males); Mean age at study:**  **13.4; Height: 159.5; Weight: 55** | **Isokinetic Quadricep Strength (Nm), handgrip strength (kg)** |
| Lim et al. 2013,  Korea [43] | **comparative study with a control group** | **40 (22 males) survivors of osteosarcoma; Mean age at study: 21.8; Height: 159.5 (females), 171.3 (males); Weight: 56.8 (females), 64.0 (males)** | **55 (26 males); Mean age at study: 19; Height: 162 (females), 175 (males); Weight: 56.5 (females), 69.1 (males)** | **Lean mass (kg) from DXA** |
| Bianco et al. 2014,  Italy [55] | **case-control** | **18 (males NG) survivors of acute leukemia and lymphoma; Mean age at study: 7.6; Height:144.6; Weight: 41.8** | **40 (males NG); Mean age at study:**  **7.92; Height: 140.6; Weight: 37.4** | **Standing broad jump (cm), Sit-up test (reps), handgrip strength (kg)** |
| Akyay et al. 2014,  Turkey [56] | **comparative study with a control group** | **18 (10 males) survivors of childhood acute lymphoblastic leukemia; Mean age at study:**  **12.5; Height: NG; Weight: NG** | **18 (10 males); Mean age at study:**  **12.5; Height: NG; Weight: NG** | **Handgrip Strength (kg)** |
| Ambroszkiewicz et al. 2015,  Poland [32] | **comparative study with a control group** | **35 (20 males) survivors of malignant bone tumours; Mean age at study: 14.8; Height: NG; Weight: NG** | **28 (13 males); Mean age at study: 14.3; Height: NG; Weight: NG** | **Lean mass (kg) from DXA** |
| Jahnukainen et al. 2015,  Finland [37] | **cross-sectional** | **49 (49 males) survivors of childhood acute lymphoblastic leukemia; Mean age at study: 30.3; Height: 177.1; Weight: 79.4** | **55 (55 males); Mean age at study: 30; Height:**  **180.4; Weight: 84** | **Lean mass (kg) from DXA; trunk lean mass (kg) from DXA** |
| Ness et al. 2015,  United States [45] | **cross-sectional** | **365 (174 males) survivors of childhood acute lymphoblastic leukemia; Mean age at study: 28.6; 163.9 (female no CRT), 158 (female CRT); 176.1 (male no CRT), 172.9 (male CRT); Weight: 71.1 (female no CRT), 81.2 (female CRT); 87.7 (male no CRT), 87.8 (male CRT)** | **365 (191 males); Mean age at study: 28.9; Height: 164.2 (females), 178.9 (males); Weight: 74.9 (females), 87.3 (males)** | **Fat-free mass (kg) from DXA;**  **Isokinetic knee extension (Newton-meters [Nm]/kg at 60and 300°/s) and ankle dorsiflexion (Nm/kg at 30°/s and 90°/s), handgrip strength (kg)** |
| Slater et al. 2015,  United States [20] | **cross-sectional** | **119 (67 males) survivors of childhood cancer (mixed diagnoses); Mean age at study: 27.4; Height: 166.1; Weight: 68.6** | **66 (36 males); Mean age at study: 25; Height: 173.7; Weight: 73.8** | **Lean body mass (kg) from DXA, Handgrip strength (kg)** |
| Genberg et al. 2015,  Sweden [46] | **cross-sectional** | **18 (10 males) survivors of acute lymphoblastic leukemia or lymphoblastic lymphoma; Mean age at study: 27.2; Height: 167.8; Weight: 63.9** | **18 (10 males); Mean age at study: 27.4; Height:179.4; Weight: 75.0** | **Fat-free mass (kg) from DXA** |
| Boland et al.  2016,  United States [33] | **cross-sectional** | **365 (174 males) survivors of childhood acute lymphoblastic leukemia; Mean age at study: 30; Height: 168.5; Weight: 81.4** | **365 (174 males); Mean age at study: 30; Height:**  **171.9; Weight: 81.4** | **Appendicular lean muscle mass (kg) from DXA** |
| Follin et al. 2017,  Sweden [36] | **case-control** | **38 (17 males) survivors of childhood acute lymphoblastic leukemia and 17 (6 males) survivors of craniopharyngioma; Mean age at study:**  **37.5 (ALL), 34.8 (CP); Height: NG; Weight: 77.4 (ALL), 79.2 (CP)** | **27 (15 males); Mean age at study: 37.25; Height: NG; Weight: 75.45** | **Fat-free mass (kg) from DXA** |
| Fernandez-Pineda et al. 2017,  United States [58] | **cross-sectional** | **157 (89 males) survivors of bone sarcoma and 49 (19 males) survivors of soft tissue sarcoma; Mean age at study: 38 (BS), 34.7 (STS); Height: NG; Weight: NG** | **206 (108 males); Mean age at study:**  **33.1; Height: NG; Weight: NG** | **Isokinetic knee extension, dorsiflexion, plantar flexion, handgrip strength (kg)** |
| Vatanen et al. 2017,  Finland [50] | **comparative study with a control group** | **19 (8 males) survivors of**  **HR NBL (one patient with HR retinoblastoma was treated in accordance with the HR NBL protocol and was included in the study); Mean age at study: 22.7; Height: 154.1; Weight: 54.8** | **20 (9 males); Mean age at study: 22.5; Height:**  **174.8; Weight: 72.18** | **Lean mass (kg) from DXA** |
| Hartman et al. 2018,  The Netherlands [65] | **cross-sectional** | **17 (11 males) survivors of**  **AML, 26 (10 males) survivors of NBL and 28 (18 males) survivors of WT; Mean age at study: 31.5 (AML), 28 (NBL) and 28.1 (WT); Height: 174 (AML), 172 (NBL) and 177 (WT); Weight: 79.2 (AML), 73.2 (NBL) and 74.5 (WT).** | **75 (36 males); Mean age at study: 22.5; Height:**  **176; Weight: 74.6** | **Push-ups (n), Sit-ups (n), Vertical jump (cm), handgrip strength (N)** |
| Wogksch et al. 2019,  United States [25] | **cross-sectional** | **336 (180 males) childhood survivors of**  **Hodgkin lymphoma; Mean age at study:**  **38.28; Height: NG; Weight: NG** | **327 (frequency matched with survivors); Mean age at study: frequency matched with survivors; Height: NG; Weight: NG** | **Knee extension (N⋅m/kg), Hand grip strength (kg)** |
| Long et al. 2019,  Australia [41] | **comparative study with a control group** | **20 (10 males) survivors of brain cancers; Mean age at study: 20; Height: 164.1; Weight: 66.6** | **19 (9 males); Mean age at study: 20; Height:**  **176.3; Weight: 74.69** | **Lean body mass (kg) from DXA** |
| Oschwald et al. 2019,  Germany [63] | **cross-sectional** | **16 (12 males) survivors of childhood cancer (mixed diagnoses); Mean age at study: 12.9; Height: NG; Weight: NG** | **16 (12 males); Mean age at study:**  **13.0; Height: NG; Weight: NG** | **Ankle dorsiflexion strength (N)** |
| Malicka et al. 2019,  Poland [68] | **comparative study with a control group** | **71 (41 males) survivors of childhood cancer (mixed diagnoses); Mean age at study: 11.2; Height: 145; Weight: 40.2** | **85 (43 males); Mean age at study:**  **10.7; Height:142; Weight: 36.3** | **Ball throw (m), Long jump (m)** |
| Long et al. 2020,  Australia [40] | **comparative study with a control group** | **19 (9 males) survivors of childhood leukemia; Mean age at study: 19; Height: 173; Weight: 76.2** | **17 (8 males); Mean age at study: 22; Height:**  **173.8; Weight: 70.1** | **Lean body mass (kg) from DXA** |
| DeFeo et al. 2020  United States [57] | **cross-sectional** | **135 (71 males) survivors of**  **acute leukemia and lymphoma with ON and 1560 (818 males) survivors**  **acute leukemia and lymphoma without ON**  **; Mean age at study: 27.7 (ON+), 33.3 (ON-); Height: NG; Weight: NG** | **272 (130 males); Mean age at study:**  **35.1; Height: NG; Weight: NG** | **Isokinetic knee extension, dorsiflexion, handgrip strength (kg)** |
| Benzing et al. 2021,  Switzerland [64] | **cross-sectional** | **61 (36 males) survivors without CNS involvement and 17 (10 males) survivors**  **With CNS involvement; Mean age at study:**  **10.9 (non-CNS), 12.4 (CNS); Height: 144.6 (non-CNS), 146.6 (CNS); Weight: 39.5 (non-CNS), 45.4 (CNS).** | **56 (29 males); Mean age at study: 11.5; Height:147.9; Weight: 41.9** | **Sit-ups, push-ups, long jump** |
| Malhotra et al. 2021,  India [42] | **case-control** | **65 (52 males) survivors of childhood acute lymphoblastic leukemia; Mean age at study: 16.3; Height: 154.6; Weight: 50.9** | **50 (sex matched with survivors); Mean age at study: matched with survivors; Height:**  **156; Weight: 48.5** | **Lean body mass (kg) from DXA** |
| Marchese et al. 2021,  United States [67] | **cross-sectional** | **6 (3 males) survivors of childhood acute lymphoblastic leukemia; Mean age at study:**  **11.5; Height: 141.5; Weight: 46.68** | **6 (3 males); Mean age at study:**  **10.9; Height:145.7; Weight: 40.83** | **Jump Height (cm)** |
| Lambert et al. 2021,  Canada [60] | **Interventional** | **9 (3 males) survivors of childhood acute lymphoblastic leukemia; Mean age at study:**  **10.2; Height: 143.3; Weight: 46.9** | **9 (3 males); Mean age at study:**  **10.5; Height:146.0; Weight: 40.7** | **Single two-legged jump test and multiple two-legged hopping** |
| Roelofs et al. 2022,  United States [47] | **comparative study with a control group** | **24 (17 males) survivors of childhood cancer (mixed diagnoses); Mean age at study: 14.6; Height: 155.2; Weight: 46.5** | **211 (117 males); Mean age at study:**  **13.8; Height:161.1; Weight: 56.1** | **Lean body mass (kg) from DXA; arm and leg lean mass (kg) from DXA** |
| Kyriakakis et al. 2023,  UK [38] | **cross-sectional** | **16 (9 males) survivors of brain tumors; Mean age at study: 20.4; Height: NG; Weight: NG** | **16 (9 males); Mean age at study:**  **21.8; Height: NG; Weight: NG** | **Lean body mass (kg) from bioimpedance; trunk lean body mass (kg) from bioimpedance** |
| Bratteteig et al. 2024,  Norway [34] | **cross-sectional** | **157 (83 males) survivors of childhood cancer (mixed diagnoses); Mean age at study: 13.4; Height: 158; Weight: 50.3** | **113 (56 males); Mean age at study:**  **13.2; Height: 158; Weight: 47.8** | **Fat-free mass (kg) from DXA** |
| Ketterl et al. 2024,  United States [21] | **comparative study with a control group** | **151 (87 males) survivors of hematologic malignancies; Mean age at study: 26.4; Height: 162; Weight: 64.2** | **92 (49 males); Mean age at study:**  **24.5; Height: 170; Weight: 72.2** | **Lean mass (kg) from DXA** |
| Romano et al. 2024,  Italy [53] | **comparative study with a control group** | **14 (14 males) survivors of brain tumors; Mean age at study: 24.9; Height: 168; Weight: 67.9** | **14 (14 males); Mean age at study:**  **24.6; Height: 177; Weight: 76.8** | **Fat-free mass (kg) from impedance** |
| Apostolaki et al. 2025,  Greece [54] | **case–control study** | **36 (16 males) survivors of childhood cancer (mixed diagnoses); Mean age at study: 13.3; Height: 158; Weight: 59.0** | **23 (11 males); Mean age at study:**  **12.2; Height: 153; Weight: 54.0** | **Fat-free mass (kg) from bioelectrical impedance** |
| Evardsen et al. 2025,  Norway [23] | **cross-sectional** | **157 (84 males) survivors of childhood cancer (mixed diagnoses); Mean age at study: 13.4; Height: 158; Weight: 50.3** | **113 (56 males); Mean age at study:**  **13.3; Height: 158; Weight: 47.8** | **Isometric knee extension (kg), chest press (kg), handgrip strength (kg), and countermovement jump (cm)** |
| Valenzuela et al. 2025,  Spain [22] | **cross-sectional** | **126 (74 males) survivors of childhood cancer (mixed diagnoses); Mean age at study: 12.8; Height: 155; Weight: 51.4** | **427 (228 males); Mean age at study:**  **11.2; Height: 151; Weight: 43.2** | **5RM seated bench press, lateral row, seated leg press, and knee-extension (kg)** |
| NG, not given; STS, soft tissue sarcoma; BS, bone sarcoma; AML, acute myelogenous leukemia; ALL; acute lymphoblastic leukemia; NBL, neuroblastoma; WT, Wilms Tumour; CP, craniopharyngioma; HR, high risk; CRT, cranial radiotherapy; ON, osteonecrosis; CNS, central nervous system; DXA, dual-energy X-ray absorptiometry; BIA, bioelectrical impedance.  *Notes: Sample sizes may vary between total cohort size and the number of participants for specific measurement; Where original studies reported median age, height, and weight values, these have been converted to estimated means using the Wan et al, 2016 [16] formula for consistency.* *For studies that did not explicitly state the study design, they were categorized as “comparative studies with a control group” based on the presence of a control group/comparison group.* | | | | |

**Table 3** Individual risk of bias assessment for studies assessing muscle quantity and function in childhood cancer survivors

| Author, year | Selection (4) | Comparability (2) | Exposure (3) | Total (9) |
| --- | --- | --- | --- | --- |
| Hovi et al. 1993,  Finland [59] | **2** | **2** | **2** | **6** |
| Talvensaari et al. 1995,  Finland [48] *(function)* | **3** | **2** | **2** | **7** |
| Talvensaari et al. 1995,  Finland [48] *(quantity)* | **3** | **2** | **2** | **7** |
| Wright et al., 1998,  Canada [62] | **3** | **2** | **2** | **7** |
| Rahim et al. 1999,  UK [35] | **3** | **2** | **2** | **7** |
| Warner et al. 2004,  UK [51] | **3** | **0** | **2** | **5** |
| Link et al. 2004,  Sweden [39] | **3** | **2** | **2** | **7** |
| Marinovic et al. 2005,  France [52] | **2** | **2** | **2** | **6** |
| Murphy et al. 2006,  UK [44] | **2** | **2** | **2** | **6** |
| VanBeek et al. 2009,  The Netherlands [49] | **2** | **0** | **2** | **4** |
| Ness et al. 2010,  United States [61] | **2** | **2** | **2** | **6** |
| Järvelä et al, 2010,  Finland [66] | **2** | **2** | **2** | **6** |
| Steinberger et al. 2012,  United States [19] | **3** | **2** | **2** | **7** |
| Hoffman et al. 2013,  United States [24] | **2** | **2** | **2** | **6** |
| Lim et al. 2013,  Korea [43] | **1** | **1** | **2** | **4** |
| Bianco et al. 2014,  Italy [55] | **2** | **1** | **2** | **5** |
| Akyay et al. 2014,  Turkey [56] | **2** | **2** | **2** | **6** |
| Ambroszkiewicz et al. 2015,  Poland [32] | **4** | **1** | **2** | **7** |
| Jahnukainen et al. 2015,  Finland [37] | **2** | **2** | **2** | **6** |
| Ness et al. 2015,  United States [45] *(function)* | **4** | **2** | **3** | **9** |
| Ness et al. 2015,  United States [45] *(quantity)* | **4** | **2** | **3** | **9** |
| Slater et al. 2015,  United States [20] *(function)* | **3** | **2** | **2** | **7** |
| Slater et al. 2015,  United States [20] *(quantity)* | **3** | **2** | **2** | **7** |
| Genberg et al. 2015,  Sweden [46] | **4** | **2** | **2** | **8** |
| Boland et al.  2016,  United States [33] | **4** | **2** | **3** | **9** |
| Follin et al. 2017,  Sweden [36] | **3** | **2** | **2** | **7** |
| Fernandez-Pineda et al. 2017,  United States [58] | **3** | **2** | **2** | **7** |
| Vatanen et al. 2017,  Finland [50] | **3** | **2** | **2** | **7** |
| Hartman et al. 2018,  The Netherlands [65] | **2** | **2** | **2** | **6** |
| Wogksch et al. 2019,  United States [25] | **3** | **2** | **2** | **7** |
| Long et al. 2019,  Australia [41] | **2** | **2** | **2** | **6** |
| Oschwald et al. 2019,  Germany [63] | **3** | **2** | **2** | **7** |
| Malicka et al. 2019,  Poland [68] | **3** | **2** | **2** | **7** |
| Long et al. 2020,  Australia [40] | **2** | **2** | **2** | **6** |
| DeFeo et al. 2020  United States [57] | **3** | **2** | **2** | **7** |
| Benzing et al. 2021,  Switzerland [64] | **3** | **1** | **2** | **6** |
| Malhotra et al. 2021,  India [42] | **3** | **2** | **2** | **7** |
| Marchese et al. 2021,  United States [67] | **2** | **2** | **2** | **6** |
| Lambert et al. 2021,  Canada [60] | **2** | **2** | **2** | **6** |
| Roelofs et al. 2022,  United States [47] | **3** | **0** | **2** | **5** |
| Kyriakakis et al. 2023,  UK [38] | **2** | **2** | **2** | **6** |
| Bratteteig et al. 2024,  Norway [34] | **3** | **2** | **2** | **7** |
| Ketterl et al. 2024,  United States [21] | **4** | **2** | **2** | **8** |
| Romano et al. 2024,  Italy [53] | **3** | **2** | **3** | **8** |
| Apostolaki et al. 2025,  Greece [54] | **1** | **2** | **3** | **6** |
| Evardsen et al. 2025,  Norway [23] | **3** | **2** | **2** | **7** |
| Valenzuela et al. 2025,  Spain [22] | **3** | **2** | **1** | **6** |

**Table 4** Results of test of moderators for muscle quantity and function in childhood cancer survivors

| **Test of Moderators** | **value** | **p** |
| --- | --- | --- |
| Muscle Quantity Cancer Type | 0.72 | 0.511 |
| Muscle Quantity Assessment Modality | 0.27 | 0.646 |
| Muscle Function Cancer Type | 0.17 | 0.851 |
| Muscle Function Assessment Modality | 6.93 | 0.026 |
| Body Region | 10.95 | 0.007 |

**Table 5** Meta-regression of age at assessment, time from diagnosis, cohort year, age at diagnosis, male sex, and height on muscle quantity and function in childhood cancer survivors

| Variables | | k | n | Univariable model | |
| --- | --- | --- | --- | --- | --- |
| Muscle Quantity |  |  |  | **β ± SE** | **P-value** |
|  | *Age at assessment* | 37 | 25 | -0.02 ± 0.01 | 0.092 |
|  | *Time off therapy* | 16 | 13 | -0.05 ± 0.01 | 0.016 |
|  | *Male Sex* | 35 | 24 | -0.02 ± 0.17 | 0.905 |
|  | *Height (MD)* | 21 | 17 | 0.06 ± 0.01 | 0.002 |
|  | *Weight (MD)* | 23 | 18 | 0.06 ± 0.01 | <0.001 |
| Muscle function |  |  |  |  |  |
|  | *Age at assessment** | 90 | 19 | 0.01 ± 0.01 | 0.029 |
|  | *– Upper body* | 40 | 15 | 0.02 ± 0.01 | 0.013 |
|  | *– Lower body* | 48 | 14 | 0.01 ± 0.01 | 0.215 |
|  | *Time off therapy* | 22 | 10 | -0.01 ± 0.04 | 0.723 |
|  | *Male Sex* | 94 | 20 | -0.04 ± 0.29 | 0.913 |
|  | *Height (MD)* | 53 | 14 | -0.01 ± 0.03 | 0.628 |
|  | *Weight (MD)* | 52 | 13 | -0.04 ± 0.02 | 0.168 |

β, meta-regression coefficient; n, number of studies; k, number of effect sizes; SE, standard error.

*Analyses further stratified by upper and lower body


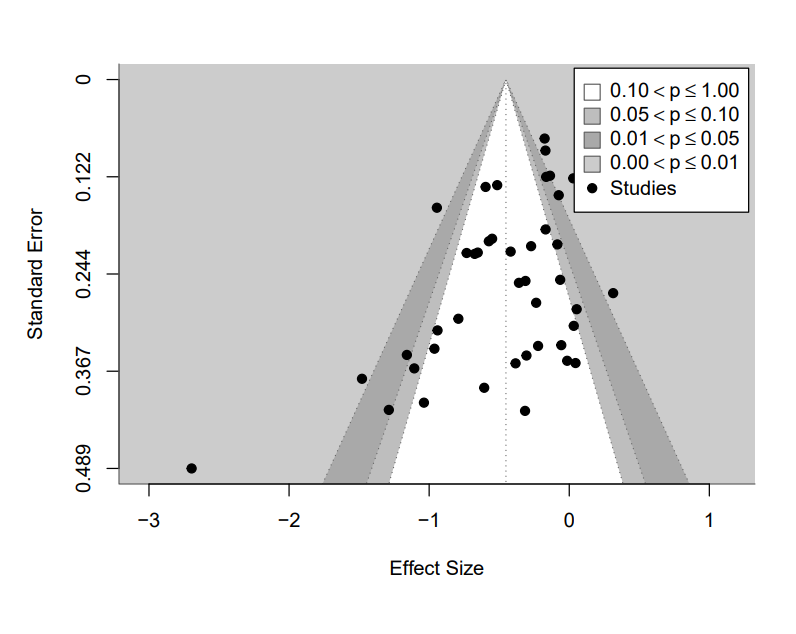


**Figure 1** Contour-enhanced funnel plot for overall effects on muscle quantity in childhood cancer survivors


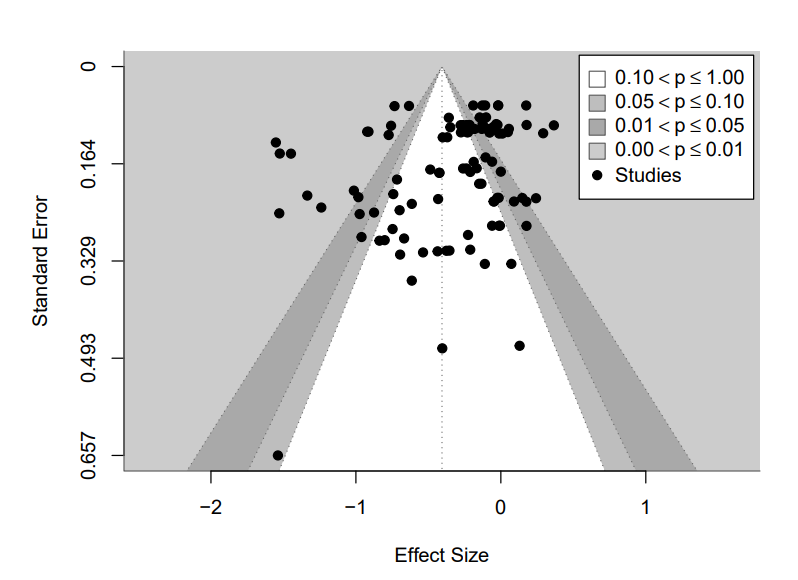


**Figure 2** Contour-enhanced funnel plot for overall effects on muscle function in childhood cancer survivors


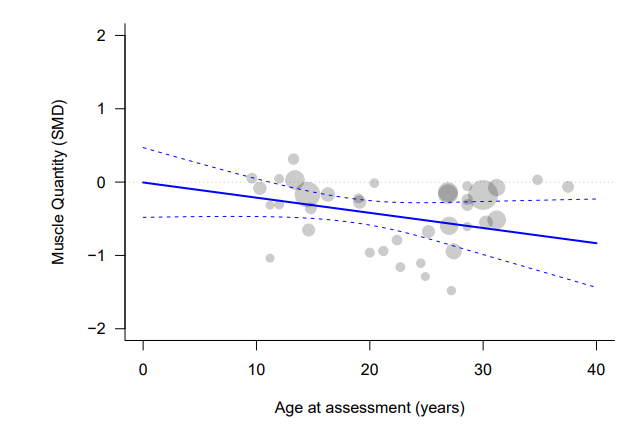


**Figure 3** Bubble plots of random effects univariable meta-regression of muscle quantity (SMD) with respect to age at assessment. Each circle represents an effect size, and the size of the circle reflects the influence of that study on the meta-regression model. The solid line represents the meta-regression slope, while the dashed represent the 95% confidence intervals


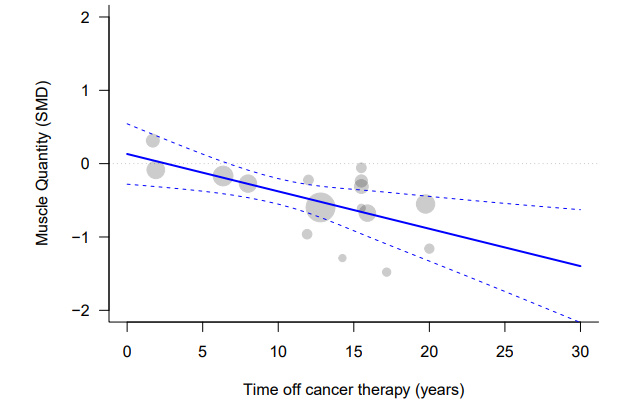


**Figure 4** Bubble plots of random effects univariable meta-regression of muscle quantity (SMD) with respect to time off therapy. Each circle represents an effect size, and the size of the circle reflects the influence of that study on the meta-regression model. The solid line represents the meta-regression slope, while the dashed represent the 95% confidence intervals


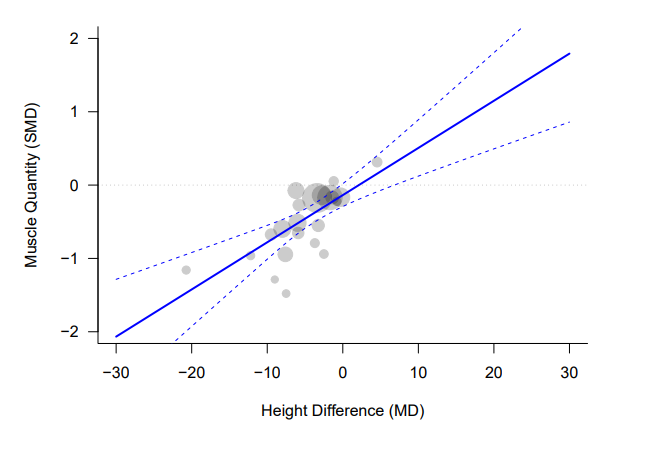


**Figure** **5** Bubble plots of random effects univariable meta-regression of muscle quantity (SMD) with respect to height (MD). Each circle represents an effect size, and the size of the circle reflects the influence of that study on the meta-regression model. The solid line represents the meta-regression slope, while the dashed represent the 95% confidence intervals


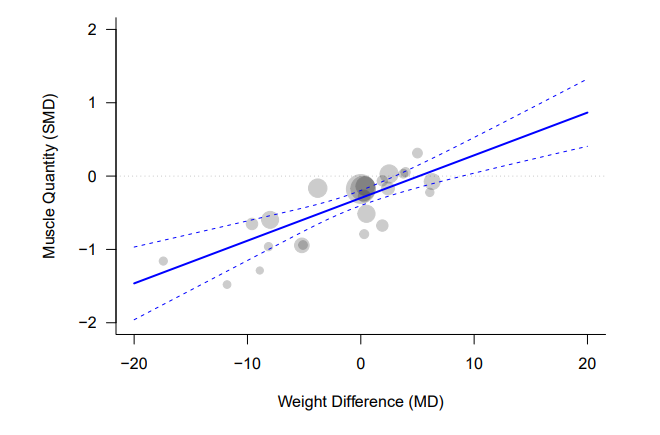


**Figure 6** Bubble plots of random effects univariable meta-regression of muscle quantity (SMD) with respect to weight (MD). Each circle represents an effect size, and the size of the circle reflects the influence of that study on the meta-regression model. The solid line represents the meta-regression slope, while the dashed represent the 95% confidence intervals

**
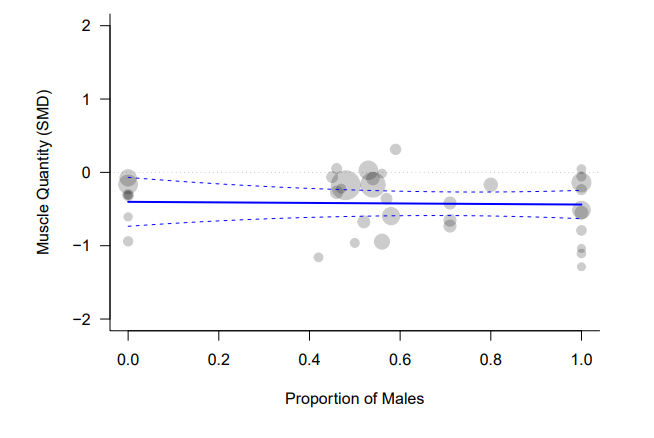
**

**Figure** **7** Bubble plots of random effects univariable meta-regression of muscle quantity (SMD) with respect to proportion of males. Each circle represents an effect size, and the size of the circle reflects the influence of that study on the meta-regression model. The solid line represents the meta-regression slope, while the dashed represent the 95% confidence intervals

**
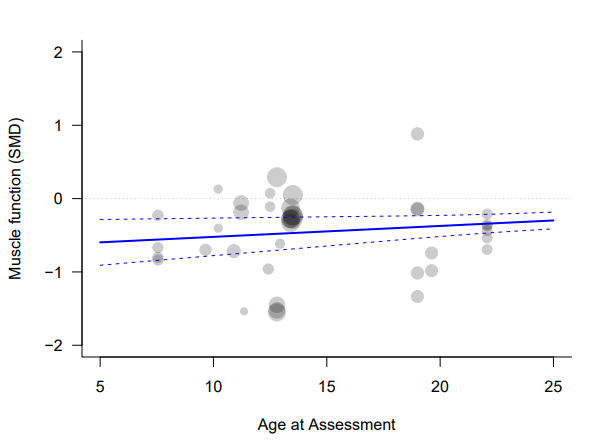
**

**Figure** **8** Bubble plots of random effects univariable meta-regression of muscle function (SMD) with respect to age at assessment. Each circle represents an effect size, and the size of the circle reflects the influence of that study on the meta-regression model. The solid line represents the meta-regression slope, while the dashed represent the 95% confidence intervals


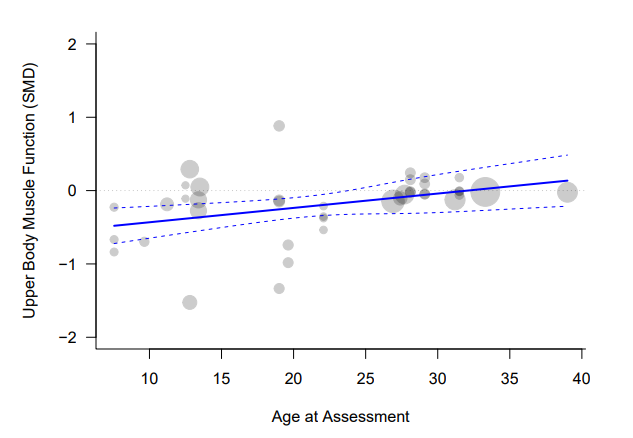


**Figure** **9** Bubble plots of random effects univariable meta-regression of upper body muscle function (SMD) with respect to age at assessment. Each circle represents an effect size, and the size of the circle reflects the influence of that study on the meta-regression model. The solid line represents the meta-regression slope, while the dashed represent the 95% confidence intervals


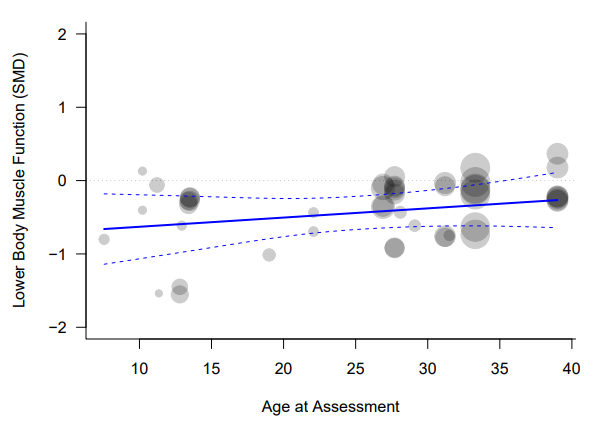


**Figure** **10** Bubble plots of random effects univariable meta-regression of lower body muscle function (SMD) with respect to age at assessment. Each circle represents an effect size, and the size of the circle reflects the influence of that study on the meta-regression model. The solid line represents the meta-regression slope, while the dashed represent the 95% confidence interval


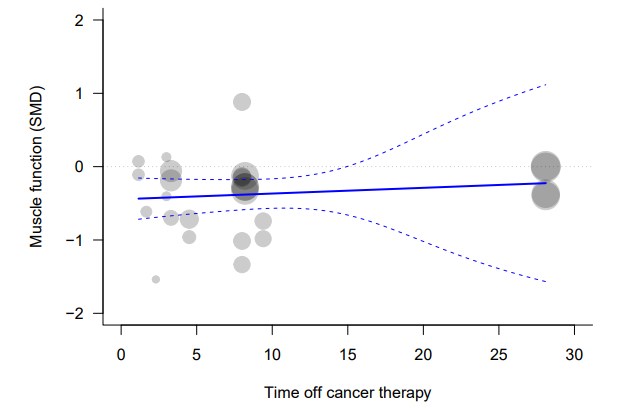


**Figure** **11** Bubble plots of random effects univariable meta-regression of muscle function (SMD) with respect to time off therapy. Each circle represents an effect size, and the size of the circle reflects the influence of that study on the meta-regression model. The solid line represents the meta-regression slope, while the dashed represent the 95% confidence intervals


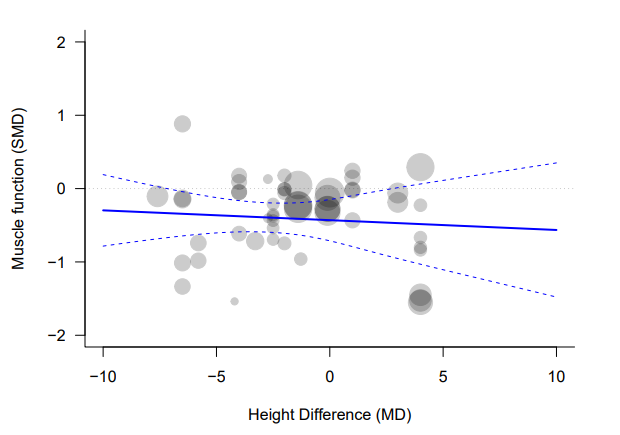


**Figure** **12** Bubble plots of random effects univariable meta-regression of muscle function (SMD) with respect to height (MD). Each circle represents an effect size, and the size of the circle reflects the influence of that study on the meta-regression model. The solid line represents the meta-regression slope, while the dashed represent the 95% confidence intervals


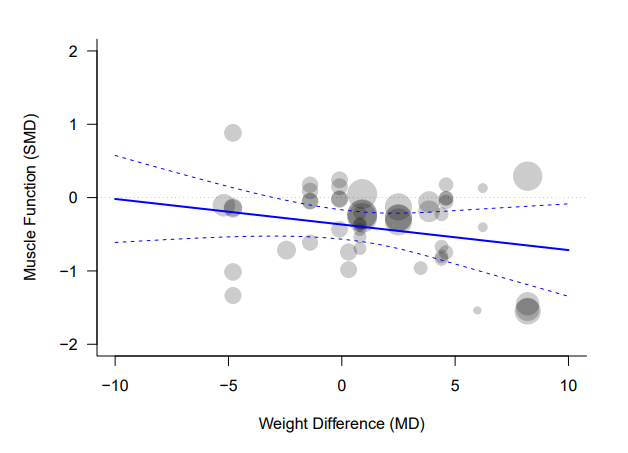


**Figure** **13** Bubble plots of random effects univariable meta-regression of muscle function (SMD) with respect to weight (MD). Each circle represents an effect size, and the size of the circle reflects the influence of that study on the meta-regression model. The solid line represents the meta-regression slope, while the dashed represent the 95% confidence intervals


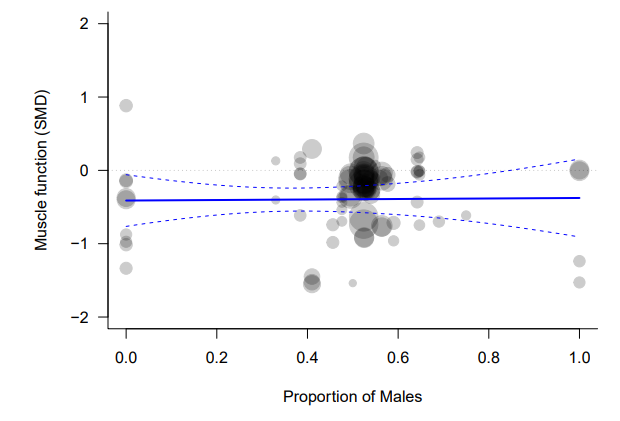


**Figure 14** Bubble plots of random effects univariable meta-regression of muscle function (SMD) with respect to proportion of males. Each circle represents an effect size, and the size of the circle reflects the influence of that study on the meta-regression model. The solid line represents the meta-regression slope, while the dashed represent the 95% confidence intervals

1. Follin, C., et al., *Improvement in cardiac systolic function and reduced prevalence of metabolic syndrome after two years of growth hormone (GH) treatment in GH-deficient adult survivors of childhood-onset acute lymphoblastic leukemia.* The Journal of Clinical Endocrinology & Metabolism, 2006. **91**(5): p. 1872-1875.

2. Polgreen, L.E., et al., *Modifiable risk factors associated with bone deficits in childhood cancer survivors.* BMC Pediatrics, 2012. **12**: p. 1-9.

3. Öberg, A., et al., *Exercise capacity in young adults after hematopoietic cell transplantation in childhood.* American Journal of Transplantation, 2018. **18**(2): p. 417-423.

4. Bülow, B., et al., *Survivors of childhood acute lymphoblastic leukaemia, with radiation‐induced GH deficiency, exhibit hyperleptinaemia and impaired insulin sensitivity, unaffected by 12 months of GH treatment.* Clinical Endocrinology, 2004. **61**(6): p. 683-691.

5. Slater, M.E., et al., *Physical activity and cardiovascular risk factors in childhood cancer survivors.* Pediatric Blood & Cancer, 2015. **62**(2): p. 305-310.
